# Exploring the Relationship Between Parental Involvement, Paper Folding Skills, and Early Spatial Ability: A Mediation Model

**DOI:** 10.3389/fpsyg.2020.568439

**Published:** 2020-12-04

**Authors:** Dandan Wu, Jin Sun

**Affiliations:** ^1^School of Education, Macquarie University, Sydney, NSW, Australia; ^2^Department of Early Childhood Education, The Education University of Hong Kong, Tai Po, Hong Kong

**Keywords:** spatial ability, folding paper, early development, parental involvement, origami

## Abstract

Paper folding is a common activity in East Asian kindergartens, but its potential value to early spatial skills have not been empirically explored. This study aims to investigate whether and how paper folding skills can predict spatial ability (SA) in the early years. Altogether 101 preschoolers (*N_girl_* = 45, *M*_age_ = 4.54, *SD* = 0.75) were randomly sampled from two Hong Kong kindergartens and invited to complete the map-use and the paper folding tasks. The paper folding task taps two levels of children’s paper folding skills: Basic Folding Skill (BFS) and Advanced Folding Skill (AFS). The parents reported the demographic information and their involvement in spatial activities at home. The results indicated the following: (1) there was a significant age-related increase in the paper folding performance; (2) child age could significantly predict both BFS (*β* = 0.551, *p* < 0.001) and AFS (*β* = 0.627, *p* < 0.001), while parental involvement could only predict BFS (*β* = 0.246, *p* < 0.001); (3) after controlling for confounders, paper folding skills could significantly predict SA as measured by the map-use task; (4) BFS was found to mediate the relationship between parental involvement and SA. The educational implications of these findings are also discussed.

## Introduction

Paper folding activity (PFA) has a long history in China (“
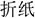
”) and Japan (‘Origami’) and has thus been listed as an Intangible Cultural Heritage of Humanity by UNESCO. It has become a popular and substantial part of Chinese and Japanese kindergartens’ learning and teaching activities (Nishida, 2019). PFA is a kind of integrated learning experience requiring young children to systematically and strategically apply their mathematic and fine motor skills; thus, it has been widely regarded as a kind of art and craft activity in the early childhood classroom. However, its potential contribution to the early development of spatial skills has not been thoroughly explored (Dinehart and Manfra, 2013; Imaroonrak et al., 2018; Widayati et al., 2019). A recent study found that paper folding skills were highly correlated with spatial ability (SA) (Taylor and Hutton, 2013), indicating that there might be a predictive relationship between them. Therefore, for the first time, this study explored the possible predictive relationships between parental involvement (PI), PFA, and early spatial skills in the context of early education in Hong Kong.

### PFA in Early Educational Contexts

Paper folding activity refers to the action of folding paper into representative shapes with some specific skills, which involves visual-motor integration, considerable cognitive effort, and a relatively competent level of mathematical conceptualization (Wenciker and Flynn, 2004; Cakmak et al., 2014; Tenbrink and Taylor, 2015; Arsl and Işıksal-Bostan, 2016). Usually, paper folding skills could be divided into two levels: (1) the basic level, which requires children to fold the paper in half equally and fold along the midline of the paper and demands fine-motor skills and visual-motor integrations (Harte and Spencer, 2014; Imaroonrak et al., 2018); (2) the advanced level, which requires children to fold the paper from multiple directions with different angles and demands the children to mentally distinguish the folding step from the next step and complete the folding as planned. The advanced level depends more on high-level cognitive functions such as movement planning (Yao and Dai, 2008) and working memory (Sato et al., 2007; Zhang, 2017). This study aimed to develop a new paper folding task including these two levels.

Paper folding activity has been considered as an origami-based problem-solving context to facilitate mathematical learning and teaching during primary to high school (Wenciker and Flynn, 2004; Cakmak et al., 2014; Tenbrink and Taylor, 2015; Arsl and Işıksal-Bostan, 2016; Oberman, 2018). Some scholars believe that origami could provide some unique mathematical experiences and thus establish the linkage between mathematics and the arts, lending varying pedagogical support to the learning and teaching of math (Wenciker and Flynn, 2004). Some even believed that paper folding could serve as a teaching tool in mathematics classes (Boakes, 2009). Turkish teachers even believed that origami might be a beneficial and effective method in primary mathematics education (Arsl and Işıksal-Bostan, 2016).

However, in the early childhood context, PFA has been widely regarded as a learning activity to develop young children’s fine motor skills and the sense of artistry (Dinehart and Manfra, 2013; Zhao, 2015; Nishida, 2019). The existing studies have widely explored its educational values on early arts and motor skills: (1) as an art education, PFA has been implemented in Japanese kindergartens for over 140 years, serving as a kind of symbolic art and craft culture (Nishida, 2019); (2) as an indicator of fine motor skills, PFA has been used to measure young children’s fine motor skills (Dinehart and Manfra, 2013; Vidoni et al., 2014; Saraiva et al., 2019); and (3) as training of visual-motor integration, PFA has been proved to significantly improve the creativity and visual-motor integration (Imaroonrak et al., 2018; Widayati et al., 2019) in young children.

Recently, STEM education has become a global concern and has been linked with PFA in the early years (Taylor and Hutton, 2013; Lippard et al., 2019). For instance, Lippard et al. (2019) regarded folding activity as a pre-engineering play in early childhood classrooms and suggested that folding activity should be considered a learning context for early engineer education (EEE). Researchers have taken SA as one of the core skills required for EEE and STEM, as empirical studies have indicated that good spatial skills significantly predict achievement in STEM (Uttal et al., 2013; Stieff and Uttal, 2015). Recognizing the correlation between PFA and SA, researchers have suggested promoting STEM education by implementing PFA in kindergartens (Taylor and Hutton, 2013; Kuhl et al., 2019). However, all these suggestions should be better justified with empirical evidence about the complicated relationships between PFA and early spatial skills.

### Spatial Ability in the Early Years

Spatial ability refers to the capacity of understanding, reasoning, and remembering the spatial relations among objects or space. It has been documented as a fundamental cognitive skill with three major constructs (Uttal et al., 2013; Mix et al., 2016; Burte et al., 2017; Rittle-Johnson et al., 2019): (1) spatial visualization, which is the ability to imagine and mentally transform spatial information; (2) form perception, which is the ability to copy and distinguish shapes from other shapes, including symbols; and (3) visual-spatial working memory, which is the ability to hold the locations of different objects, landmarks, and so on in working memory. There are significant age, gender, and individual differences in the early development of spatial skills (Voyer et al., 1995; Astur et al., 2004; Parsons et al., 2004; Newcombe, 2010; Uttal et al., 2013; Rittle-Johnson et al., 2019). For example, some scholars (Peters, 2005; Maeda and Yoon, 2013) have had different views on the gender difference in SA, and Levine et al. (2016) argued that the gender gap in SA could be bridged if there was appropriate training. Therefore, it is important to ascertain the contributors from the family and preschool to better design appropriate training programs of SA (Rittle-Johnson et al., 2019).

The gender gap in SA has triggered another debate surrounding the ‘nature-nurture controversy’ in child development (Casey, 1996; Kass et al., 1998; Hoffman et al., 2011). On the one hand, Halpern (1992) indicated that hormones could have provided men with a slight advantage to foster SA, driving them to be willing to engage in related activities and reinforcing their SA from infancy to adulthood. On the other hand, Kass et al. (1998) argued that strong social encouragement to engage both boys and girls in spatial tasks could help narrow the gender gap in SA. Therefore, Tosto et al. (2014) conducted a comparison study with 4,174 pairs of 12-year-old twins and found that the environmental factors explained about 67% of the variation in SA, implying that SA could be ‘nurtured.’ Very recently, however, Rimfeld et al. (2017) duplicated the study of twins but found a greater effect of a genetic component on general SA (69%) than the environmental component (23%). They concluded that the genetic contribution to SA was generated from various kinds of genes, each making a small contribution. In Chinese children, studies have also identified significant gender differences in early SA. For example, Seng and Tan (2002) found there were cultural and gender differences in spatial abilities. Chan (2007) found differentiated gender differences: there were modest gender differences in visual arts favoring girls, while there were variations in visual orientation favoring boys. These mixed results have raised more questions in terms of how SA could be nurtured in the school and family contexts, which will be explored in this study with the newly developed map-use task. In particular, this study was focused on the relationship between PI in young children’s spatial-related activities at home and children’s performance on the PFA and map-use tasks.

In this study, the map-use task was developed from the one designed by Bluestein and Acredolo (1979) to evaluate early spatial skills for the following reasons. First, it is technically challenging to measure SA in young children because there is a lack of consensus on the definition and age-appropriate content (Rittle-Johnson et al., 2019). Second, two challenging problems should be solved before designing the age-appropriate measurement: (1) how to incorporate all the domains of SA into one single indicator, as different spatial tasks could only gauge different aspects of SA (Rittle-Johnson et al., 2019); and (2) how to make it workable with young children, as the existing spatial measures include paper-and-pencil tasks, the manipulation of objects, or computer-based tasks that are not applicable for young children (Ilen, 2016). Therefore, some scholars have tended to use the map-use task to evaluate young children’s SA (Blades and Spencer, 1986; Freundschuh, 1990; Liben et al., 2013). Third, the map-use task mainly evaluates the ability to locate places in the room, to indicate one’s own position in the room, to plan routes on maps, and so on. All these abilities could reflect (and would be affected by) the spatial visualization and spatial working memory, the two major constructs of SA (Gilmartin and Patton, 1984; Blades and Spencer, 1986; Sandberg and Huttenlocher, 2001). Finally, the existing studies by Blades and Spencer (1986) have confirmed that this task could apply to 3- to 5-year-old children. Therefore, this map-use task was revised and adopted in this study.

### Folding Activity, Spatial Ability, and Parental Involvement

The relationship between folding activity and SA has been explored from two divergent perspectives: (1) folding activity supports SA; and (2) folding activity is integrated into SA. In particular, the first view has been widely employed to study early spatial development. For example, the studies on young Japanese and American children (Yuzawa et al., 1999), middle school students (Boakes, 2009), and primary students (Cakmak et al., 2014) have jointly confirmed the first view that folding activity could improve SA. Yuzawa and Bart (2002) specifically noted that the experience of origami facilitated young children’s spatial learning such as size comparison. However, the second view was also supported by many psychologists who tended to use the concept ‘mental folding skill’ to reflect a certain aspect of SA (Milivojevic et al., 2003; Wright et al., 2008; Harris et al., 2013). For example, both Milivojevic et al. (2003) and Wright et al. (2008) employed mental folding skills as an indicator of spatial skills in adults. Harris et al. (2013) developed a mental folding test and found it applicable and reliable for young children. The mental folding task in these studies, however, mainly involved evaluating specific spatial skills (i.e., spatial transformation), leaving out most of the other domains in a physical PFA such as visual-motor integration. Thus, it should not be regarded as equivalent to the typical PFA in an early childhood setting. In this study, PFA is not a mental folding skill but a physical activity to fold papers into the target figure, which may correlate with spatial skills. Therefore, this study explored whether PFA predicts early SA.

Parental involvement has been documented to have a significant impact on children’s development and later academic achievement (Fan and Chen, 2001; Jeynes, 2006, 2007; Lomax-Bream et al., 2007; Lau et al., 2012; Castro et al., 2015). According to Jeynes (2006), East Asian kindergartens, as faithful practitioners of the Froebel model, have greatly promoted PI in early educational practices. Therefore, considering PI in early SA development is suitable for the Hong Kong context (Jeynes, 2006; Lau et al., 2012). However, little is known about whether PI in spatial-related activities can enhance children’s SA and whether children’s performance on PFA can play a role in this relationship. There has been no consensus on the relationship between PI and SA due to the nature-versus-nurture debate of SA (Casey, 1996; Kass et al., 1998; Hoffman et al., 2011). Some researchers have held the belief that SA is predetermined by nature, and nurturing factors, such as PI, might thus play non-significant roles (Halpern, 1992). In contrast, some other scholars believed that SA could be influenced by educational factors including PI (Tosto et al., 2014). The recent study by Rimfeld et al. (2017) indicated that both the natural base and the environmental components during the nurturing process could contribute to the development of SA, implying that the effects of PI on SA might not be so direct. In addition, the link between PI and folding skills has been rarely explored. Therefore, this study is dedicated to exploring whether and how PI could predict children’s SA through the potential mediation of folding paper.

### The Current Study

The literature review has indicated the following relationships among PI, folding skills, and SA: (1) folding skills in early years may be influenced by PI (Fan and Chen, 2001; Jeynes, 2007; Castro et al., 2015); (2) folding skills might correlate with the SA (Boakes, 2009; Cakmak et al., 2014); and (3) PI might influence the SA, while the effect of which might not be direct (Tosto et al., 2014; Rimfeld et al., 2017). Theoretically, it is reasonable to hypothesize that the effect of PI on SA might be mediated by paper-folding skills. Therefore, an empirical exploration is needed urgently to test this hypothesis.

To achieve this end, first, this study has developed a paper folding task and analyzed its reliability and constructs with Chinese preschoolers. The malleability of paper folding skills was also examined with a focus on age and gender differences in the early years. It particularly ascertained whether the widely reported age and gender effects could be found in the two levels of paper folding tasks. Second, this study has also explored parent involvement’s influences on early folding performance with those confounding variables being controlled for. Last, the predictive relationships among PI, paper folding performance (PFP), and spatial skills were investigated using a mediation model. In particular, the following four research questions guided this study:

(1) What are the reliability and potential constructs of the paper folding task newly developed in this study?
(2) Are there any age and gender differences in the folding performance in Hong Kong preschoolers?
(3) How does PI predict early folding performance after controlling for age, gender, and family SES?
(4) Does PFP mediate the relationship between PI and spatial skills?

## Materials and Methods

### Participants

This study was part of a larger study examining early child development in Hong Kong. Altogether 101 children (*N_girl_* = 45) aged from 3.08 to 5.92 (*M*_age_ = 4.54, *SD* = 0.75) were randomly sampled from two kindergartens in Hong Kong. Both kindergartens were non-profit-making organizations, providing whole-day and half-day programs with the same story-based curriculum. The Story Approach of Integrated Learning is the dominant curriculum widely used by most of Hong Kong kindergartens, allowing teachers to intergrade different learning activities into an interesting story (Li and Chau, 2010; Li et al., 2012). All the participating children were right-handed and not diagnosed with any developmental delay. The research consent forms were signed and obtained from the principals and parents in advance of data collection. Before the formal test, the first author observed and trained the participants to confirm whether they have experience of doing similar tasks. Only the children without previous exposure to the tasks were included in this study.

### Measures

#### Map-Use Task

The map-use task was adapted from the classical experimental task developed by Bluestein and Acredolo (1979), who asked young children to identify the pictures on the map and point to the referents accordingly. This map-use task is a comprehensive test of the three constructs of young children’s SA (Mix et al., 2016; Burte et al., 2017; Rittle-Johnson et al., 2019): (1) spatial visualization is the ability to imagine and mentally transform spatial information; (2) form perception is the ability to copy and distinguish shapes from other shapes, including symbols; and (3) visual-spatial working memory is the ability to hold the location information of different objects, landmarks, etc. in working memory. The Cronbach’s Alpha for all the four scoring items for map-use skills was 0.67, indicating acceptable reliability.

**FIGURE 1:**
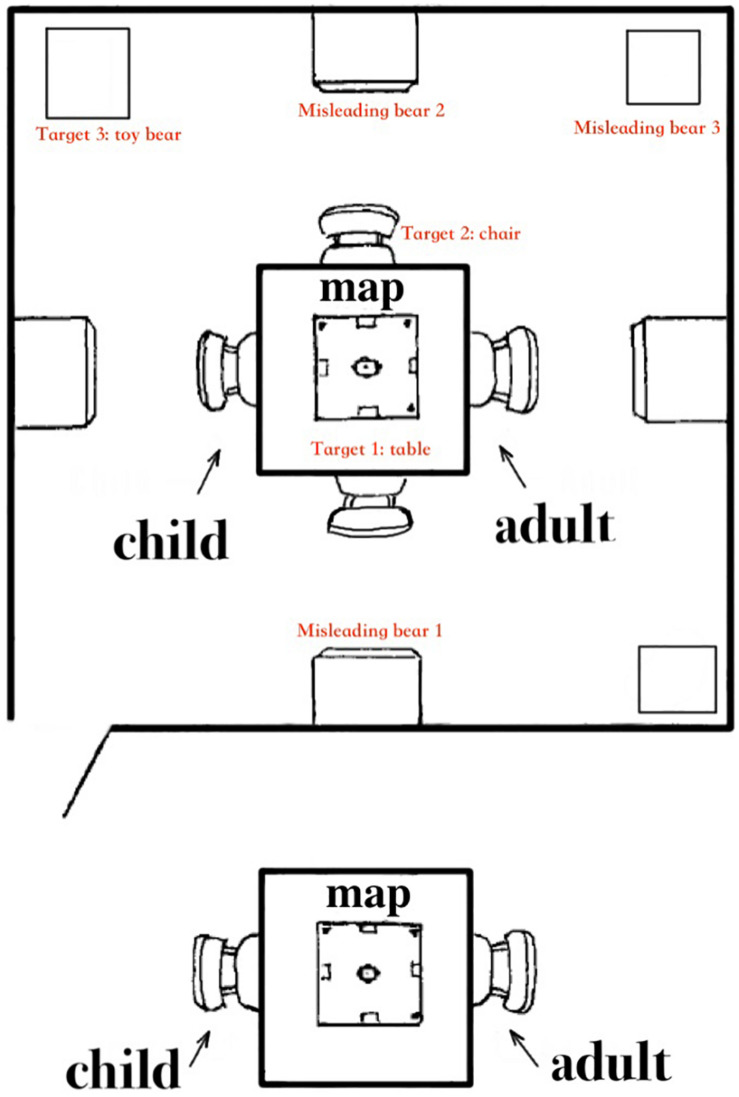
The setting of the map-use task.

The map-use test was conducted in the setting, as shown in Figure 1. The experimenter instructed the participating child as follows: “*This is the map of this room. Please have a look at this map: you are here, and the bear is over there. Please, according to this map and find the toy bear in the room.*” It was conducted during individual sessions with one experimenter following the same procedure and protocol as follows.

Step 1: the child was guided by the examiner to walk around in the room starting from the door, while the examiner introduced the major referents in the room matching with the map (the door and the equipment).
Step 2: the child was asked to read the map and to point out the location of the testing table in the room (scoring item 1), which mainly required the child to have the cognitive foundation of visual-spatial working memory to hold the location information of different objects and landmarks in mind and to recognize them.
Step 3: the child was asked to point out the location of the particular chair in the room (scoring item 2), which mainly demanded spatial visualization so that the child could imagine and mentally match the spatial information in the room with that on the map.
Step 4: the child was asked to stand outside the room and then find the toy bear as indicated by the map. In this step, three similar toy bears were placed in the room, including the target one and three distracting ones, to control the chance probability. When the setting was ready, the child was asked to return to his seat in the room, look at the map on the table, and go to find the toy bear (scoring item 3). To complete the task, the child’s form perception was mainly involved in this step, which facilitates the child to copy and distinguish the targeted symbol on the map from the misleading ones.
Step 5: two separate goals were contained in this step, including the child’s behavioral result of getting the right bear and the child’s correct reflection about this behavior. After the child got the toy bear, the experimenter asked the child to reflect whether he or she got the right bear as indicated on the map (scoring item 4). If the child answered no, he or she would then be given a second chance to find the toy bear. Then, the examiner would repeat the question asking young children to confirm whether the bear was taken from the target place marked on the map. When the child doubted his or her choice in the second time, the task was terminated, and the performance of the second time would serve for scoring. In this step, there were possible four levels of performance: (1) the child got the wrong bear but did not know it was wrong; (2) the child got the right bear but doubted his or her choice; (3) the child got the wrong bear, and realized it was wrong; (4) the child got the right bear and confirmed his or her choice. More specifically, level (1) shows that the child cannot accomplish the two separate goals, while level (4) indicates two accomplishments, and level (2) or level (3) demonstrates only one accomplishment.

The scoring process started from step 2 (scoring item 1) when the child executed the task and ended at step 5 (scoring item 4), resulting in a maximum score of 5. Specifically, from step 2 to step 4, one point was scored for each step completed, while zero point was scored if the child failed to complete that step. For step 5, different points were scored for the four levels of performance: zero points for level (1), one point for level (2) or level (3), and two points for level (4).

#### Paper Folding Task

**FIGURE 2:**
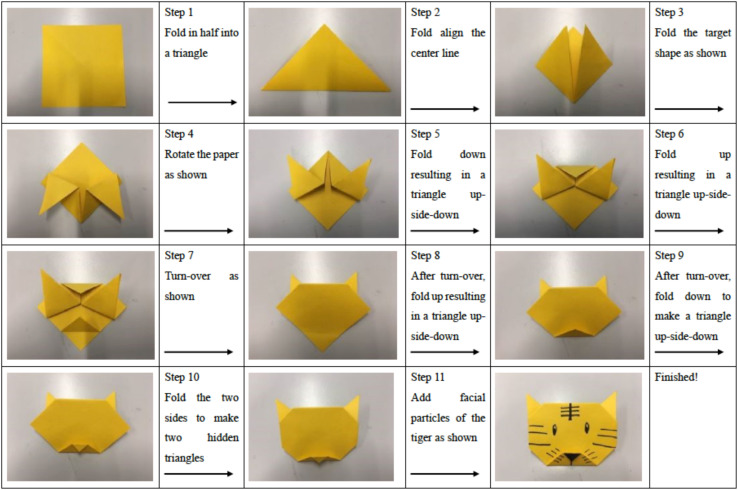
The folding task (folding a tiger).

In this study, we developed the paper folding task to examine children’s PFP based on the two criteria: first, it should be equal to the daily folding activity in kindergartens (aged 3–5), involving the fine motor skill and visual-motor integration; second, it should involve different levels of folding competence. Accordingly, ‘folding a paper tiger,’ similar to one of the most popular paper folding tasks ‘folding a paper plane’ in Chinese and Japanese kindergartens, was developed for this study (see Figure 2). With the help of both the verbal instructions provided by the experimenter and the demonstrative flow diagram, the participating child went through 11 steps to take different folding actions, which could be classified into two levels of folding performance: the basic level (Basic Folding Skills, BFS) and the advanced level (Advanced Folding Skills, AFS).

As shown in Figure 2, BFS includes three basic folding steps and skills: Step 1 involved folding in half into a triangle; Step 2 involved folding to align the centerline; and Step 3 involved folding the target shape as shown. AFS includes the following folding actions and skills: Step 4 involves rotating the paper as shown; Step 5 involves folding down, resulting in an upside-down triangle; Step 6 involves folding up, resulting in an upside-down triangle; Step 7 involves turn-over as shown; Step 8 involves, after the turn-over, folding up, resulting in an upside-down triangle; Step 9 involves, after turn-over, folding down to make an upside-down triangle; Step 10 involves folding the two sides, resulting two hidden triangles; and Step 11 involves adding the facial characteristics of the tiger as shown. For each step, the child was allowed to have one chance to receive a cue or prompt given by the examiner. If the child failed to complete a certain step even after receiving a cue, the task would end. For each step completed, the child gets two points for successful completion without prompts, 1 point for successful completion with prompt, and 0 for failing to complete. The reliability and construct validity of this task were examined.

#### Parent Survey

The parents of participating children were invited to complete a parent questionnaire, which aimed to survey the demographic information and PI. The demographic information part included the monthly household income and education degrees of the parents. The PI part used a five-point Likert scale containing eight items to evaluate the frequency of parent-child activity related with SA: how often do you (1) do crafts with your child; (2) read or use a map with your child; (3) teach your child spatial relations with the reference of his or her own body; (4) teach your child spatial relations with the reference of other objects; (5) teach your child to recognize, compare and name the shapes; (6) teach your child to remember or describe the routes from home to school; (7) ask your child to guide you to somewhere familiar/playing blocks or puzzles together; and (8) play puzzle or block building with your child? The Cronbach’s Alpha of the survey was 0.84 showing good reliability of the scale.

### Procedures

All the tasks were administered in a classroom within the kindergartens that participants were familiar with. One examiner conducted all the tasks for each participant individually. It took a total of 15–20 min on average for each participant to complete the two tasks (5–10 min per task). Before the formal task, the first author (the examiner) invited each participant to ‘participate in classroom play’ and briefed them about the related information. After the participant settled down, the examiner instructed the tasks’ rules and encouraged the participant to complete the task as required. To avoid the order bias, for each of the two kindergartens, half of the participants conducted the task by order of map-use task first and then the folding task, and the other half of them conducted the task in the opposite order. Participants were allowed to quit during the task for any reason.

### Data Analysis

First, the reliability and construct validity of the paper folding task were examined using the factor analysis. Second, the age and gender effects in the map-use and paper folding tasks were explored by MANOVA analysis with age (3) and gender (2) as independent variables and SA and folding skills as dependent variables. Third, the relationships between the study variables were explored using the correlation analysis. Fourth, the possible contributors to young children’s folding performance and its predictive power of map-use performance were investigated by two sets of hierarchical regression analyses. Last, based on the above analyses, a bootstrapping analysis using IBM SPSS Statistics version 23.0 and macro-program PROCESS 3.2 was conducted to test the mediation effect of the paper-folding performance. The bias-corrected bootstrap method with 5,000 resamples was employed to calculate the 95% confidence intervals (CI).

## Results

### Reliability and Exploratory Factor Analysis (EFA) of the Folding Task

The Cronbach’s Alpha for all the 11 folding steps was 0.92, indicating excellent reliability. Principle component analysis was conducted on the sample to explore the construct validity of the folding task. First, the adaptability of the predicted data was tested, and the results indicated that the data were suitable for exploratory factor analysis, KMO = 0.914, Bartlett spherical test *χ^2^* = 664.748 (*df* = 55, *p* < 0.001). Second, Principal Component Analysis with the Varimax rotation method yielded a two-factor model for the folding task, which could explain 9.81 and 56.04% of the variance, respectively, accounting for 65.86% of the total variation (see Table 1). The eigenvalues for the two constructs were 1.08 and 6.17. The factor loadings of the two constructs ranged between 0.62 and 0.87, and no cross-loading was above 0.30. These results indicated that the newly designed folding task could be used for the targeted sample with the two-level constructs of BFS and AFS.

**TABLE 1:** Exploratory factor analysis and confirmatory cluster structures for the paper folding task.

**Item**	**Factor1**	**Factor2**
**Basic Folding Skill**		
Step 1	0.870	
Step 2	0.802	
Step 3	0.667	
**Advanced Folding Skill**		
Step 4		0.683
Step 5		0.723
Step 6		0.624
Step 7		0.710
Step 8		0.682
Step 9		0.785
Step 10		0.791
Step 11		0.710
Eigenvalue	1.079	6.165
Explained Variance	9.814%	56.041%
Total Explained Variance	65.855%	

*KMO = 0.914; Approx. Chi-Square (*df*) = 664.748 (55), *p* < 0.001.*

### Age and Gender Differences in Folding Performance and Spatial Ability

First, the descriptive analysis showed that there was an increasing trend in folding performance from age 3–5 (*M*_aged 3_ = 7.154; *SD* = 5.583; *M*_aged 4_ = 14.625; *SD* = 5.504; *M*_aged 5_ = 18.086; *SD* = 3.293) and a growing trend in SA of the participating preschoolers (*M*_aged 3_ = 2.039; *SD* = 1.455; *M*_aged 4_ = 3.200; *SD* = 1.548; *M*_aged 5_ = 3.943; *SD* = 1.130) (Table 2). Second, MANOVAs was employed to examine the age and gender effects as well as age × gender effects in both the SA and folding skills. The results showed that there were significant age effects in both SA (*p* < 0.001) and folding skills (*p* < 0.001). In contrast, no significant gender effects or age × gender effect were found for either tasks (*p*_s_ > 0.05). Specifically, for the SA, the *Post Hoc* Tests indicated that there were significant age differences in early SA between children aged 3 and 4 (*p* < 0.01), and between children aged 3 and 5 (*p* < 0.001), but no significant age difference was found between children aged 4 and 5. For the folding skills, the *Post Hoc* tests showed that for the AFS level, a significant age difference was found between each two age groups (*p*_s_ < 0.001). However, there were no significant age differences between the 4-year-olds and the 5-year-olds for the BFS level. All these results jointly indicated a significant age difference in the PFP, while the 4-year-old and 5-year-old children had no performance differences at the BFS level.

**TABLE 2:** Mean, SD, and age difference in the paper folding and map-use tasks.

	**3;6**		**4;6**		**5;6**			
	***N* = 26**		***N* = 40**		***N* = 35**			
**Task**	***Mean***	***SD***	***Mean***	***SD***	***Mean***	***SD***	***F***	***p*-Value**
Map-use	2.039	1.455	3.200	1.548	3.943	1.130	14.001**	0.000
Folding (Whole)	7.154	5.583	14.625	5.504	18.086	3.293	38.230**	0.000
BFS	3.654	1.917	5.400	1.105	5.829	0.382	25.967**	0.000
AFS	3.500	4.188	9.225	4.875	12.257	3.128	33.353**	0.000

**p < 0.001. BFS, Basic Folding Skills; AFS, Advanced Folding Skills. *Post Hoc* Tests indicated no significant age difference between the 4-year-old and 5-year-old children during the BFS part (*p* = 0.284).

### Hierarchical Regression Analyses Predicting Paper Folding Performance

First, to explore the variables associated with early folding performance, we conducted Spearman correlation analysis on the variables involved (Table 3). The correlation matrix indicated that there were significant positive associations between folding performance and the following factors: map-use skills (*r* = 0.505, *p* < 0.01) and child age (*r* = 0.644, *p* < 0.01). Next, to explore the possible predictors of the two levels of folding performance in the early years, we entered the child age, household income, parents’ educational levels, and PI in the three-step hierarchical regression model (Table 4). The results showed that: (1) child age could significantly predict both Basic (*β* = 0.551, *p* < 0.001) and Advanced (*β* = 0.627, *p* < 0.001) levels of folding; (2) and PI only predicted the BFSs in early years but could not predict the variation in AFSs (*β* = 0.246, *p* < 0.001). This finding indicated that PI might play a vital role in developing children’s BFSs in the early years.

**TABLE 3:** Correlations among the study variables.

	**1**	**2**	**3**	**4**	**5**	**6**	**7**	**8**
(1) Map	–							
(2) Folding	0.505**	–						
(3) Parent Involvement	0.118	0.184	–					
(4) Child Age	0.467**	0.644**	−0.041	–				
(5) Child Gender	0.072	0.011	−0.042	0.078	–			
(6) Mother Education	0.047	−0.071	0.154	−0.072	0.096	–		
(7) Father Education	−0.02	−0.004	−0.021	−0.058	0.121	0.493**	–	
(8) Household Income	−0.014	−0.036	−0.01	−0.072	−0.092	0.546**	0.291**	–

N = 101. **p < 0.01 (two-tailed).

**TABLE 4:** Summary of hierarchical regression analyses predicting paper folding skills.

	**Level 1: Basic Folding Skills**				**Level 2: Advanced Folding Skills**			
	***β***	***R*^2^**	***ΔR^2^***	***F for models***	***β***	***R*^2^**	***ΔR^2^***	***F for models***
Step 1		0.302	–	21.186***		0.391	–	31.446***
Child gender	−0.050				−0.033			
Child age	0.551***				0.627***			
Step 2		0.305	0.003	8.331***		0.395	0.004	12.411***
Household income	0.057				0.144			
Father education	0.030				0.734			
Mother education	−0.047				−0.616			
Step 3		0.362	0.057	8.895***		0.409	0.013	10.825***
Parent involvement	0.246**				0.119			

**P < 0.01; ***P < 0.001.

### Path Analysis of Parental Involvement, Two-Level Folding Performance, and Spatial Ability

First, to determine the predictive power of paper-folding performance to the SA in the early years, we conducted four-step hierarchical regression analyses with map-use skills as the dependent variable. The results are shown in Table 5. In Step 1, we entered age and gender to control for their effects. In Step 2, household income, father’s education level, and mother’s education level were entered. In Step 3, we entered PI to control for its effects. In Step 4, the folding performance was entered by full folding skills (FS), BFS, and AFS, respectively. The change in *R*^2^ between the four steps indicated that (1) the children’s age and gender could jointly explain 21.9% of the variation in map-use performance. Additionally, age was found to be the most significant predictor of this SA; (2) household income, father education, and mother education could jointly explain 0.8% of the variation in children’s performance in using a map. However, none of them was a significant predictor; (3) parent involvement could only explain 1.4% of the variation in children’s map-use skills. However, it was not the significant predictor of the map-use skills; (4) full folding skills as the significant predictor of the map-use skills could explain 6.6% of the variation in map-use skills, while, specifically, BFS (6.1%) could explain more variation in map-use skills than the AFS (5.2%). The findings indicated that folding performance could serve as a significant predictor of SA when controlling for child age, gender, SES, and PI.

**TABLE 5:** Summary of hierarchical regressions predicting spatial ability (map-use).

	**Beta**	***R*^2^**	***ΔR^2^***	***F***		**Beta**	***R*^2^**	***ΔR^2^***	***F***		**Beta**	***R*^2^**	***ΔR^2^***	***F***
Step 1		0.219	–	13.759***	Step 1		0.219		13.759***	Step 1		0.219		13.759***
Gender	0.036				Gender	0.036				Gender	0.036			
Age	0.464***				Age	0.464***				Age	0.464***			
Step 2		0.227	0.008	5.593***	Step 2		0.227	0.008	5.593***	Step 2		0.227	0.008	5.593***
SES	−0.028				SES	−0.028				SES	−0.028			
Dad Edu	−0.045				Dad Edu	−0.045				Dad Edu	−0.045			
Mom Edu	0.116				Mom Edu	0.116				Mom Edu	0.116			
Step 3		0.242	0.014	4.993***	Step 3		0.242	0.014	4.993***	Step 3		0.242	0.014	4.993***
PI	0.123				PI	−0.123				PI	−0.123			
Step 4		0.308	0.066	5.901***	Step 4		0.303	0.061	5.774***	Step 4		0.294	0.052	5.531***
FS	0.344***				BFS	0.310**				AFS	0.297*			

N = 101. *p < 0.05; **p < 0.01; ***p < 0.001. Dad/Mom Edu, Father/Mother education degree obtained; PI, parental involvement in teaching children’s spatial knowledge in daily life; FS, folding skills; BFS, basic folding skills; AFS, advanced folding skills.

**FIGURE 3:**
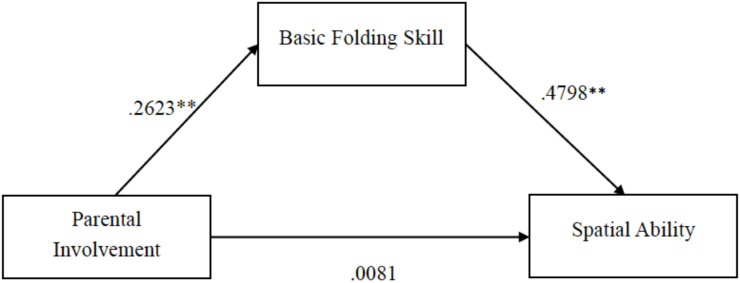
The confirmed mediation model.

Second, based on the literature review and the correlation matrix, we conducted the mediation analysis using the Bootstrap (model 4, sampling 5000 times) method to examine the direct and indirect effects of PI and Paper Folding Performance (PFP, BFS, and AFS, respectively) on SA. As shown in Table 6, the results indicated that in the significant full model: (1) PI had no significant direct influence on SA (*β* = 0.0081, 95% CI ranged from −0.1744 to 0.1906); but (2) the indirect effect of PI → BFS → SA was significant (*β* = 0.4798, 95% CI ranged from −0.2511 to −0.0299). No significant results were found in other paths and other indirect effects (see Table 6). All these findings jointly support the mediating role of BFS in this model in which PI indirectly influenced SA through BFS. The final model for this sample is presented in Figure 3.

**TABLE 6:** Direct and indirect effects of parental involvement on spatial ability in early years.

**Paths**	**Full**	
	**Effect**	**95% CI**
*Paper Folding Performance as Mediator*		
Parental Involvement → Spatial Ability	−0.0400	[−0.3149, 0.2349]
Parental Involvement → Paper Folding Performance →Spatial Ability	−0.1439	[−0.3298, 0.0067]
*Basic Folding Skill as Mediator*		
Parental Involvement → Spatial Ability	0.0081	[−0.1744, 0.1906]
Parental Involvement → Basic Folding Skill →Spatial Ability	−0.1259	[−0.2511, −0.0299]
*Advanced Folding Skill as Mediator*		
Parental Involvement → Spatial Ability	−0.0479	[−0.2260, 0.1302]
Parental Involvement → Advanced Folding Skill →Spatial Ability	−0.0698	[−0.1847, 0.0197]

*All the raw scores were transformed into *z* score to indicate Parental Involvement level, spatial ability, and paper folding performance.*

## Discussion

The primary objective of this study was to explore the predictive relationship between PI, PFP, and SA in Chinese preschoolers. The results indicated a mediation of BFS between PI and early spatial skills.

### Developmental Patterns of Paper Folding Skills and Spatial Skills

This study has developed and validated a paper folding task that could be used for young children aged 3–5. Factor analysis results have yielded two constructs: the BFS and the AFS. The psychometric results indicated that it has satisfactory reliabilities and construct validity thus could be used as a reliable measure to evaluate young children’s paper folding skills. This two-construct model is consistent with the existing studies (Wenciker and Flynn, 2004; Cakmak et al., 2014; Tenbrink and Taylor, 2015; Alebna et al., 2016).

First, this study found a significant age difference in both BFS and AFS, indicating a developmental trend of paper folding skills in the early years. This finding suggests that folding skills are malleable during the preschool years and develop from age 3 to 5. However, no significant age-related increase was found between children aged 4 and 5, suggesting that children may acquire the BFS at age 4 and maintain it to age 5. In contrast, a significant age-related increase was found in AFS between age 4 and age 5, indicating that the AFS might still develop during the 2 years. Along with the development of folding skills, significant age effects were also found in the map-use performance, implying a developing trend of SA during early childhood. Nevertheless, all these findings have jointly indicated an age-related increase in the early years, providing sound evidence to support the malleability of both abilities (Taylor and Hutton, 2013; Lippard et al., 2019).

Second, this study found no significant gender differences in both paper folding and map-use performance. This finding has provided empirical evidence to challenge the belief that spatial abilities should be biologically determined by gender-related hormones (Halpern, 1992; Rimfeld et al., 2017). This finding, however, is inconsistent with that reported by Seng and Tan (2002) and Chan (2007), who both found some significant gender differences in SA. This discrepancy might be caused by the differences in the spatial tasks, indicating that more empirical studies with consistent measures and tasks should be conducted to further explore the gender differences in SA.

### Predictors of Paper Folding Performance

This study found that PI could predict the variation in BFSs, after controlling for age and gender. This finding indicated that PI might play a critical role in developing young children’s BFSs (instead of advanced skills) in the early years. This is consistent with the existing studies that have found that interactive parenting enhanced children’s fine motor skills (Gutman and Feinstein, 2010). Other studies have also found that parenting behaviors could predict young children’s cognitive development (Rubin et al., 2002). The PFA requires the integrated involvement of both cognitive and fine motor skills; thus, it should be affected by PI, as found in this study. However, the impact of PI on PFP could only be found in developing basic skills. Those advanced skills in PFP could not be predicted by PI, indicating that there might be some intrinsic or even genetic factors contributing to its development. This possibility, however, cannot be ruled out in this study, warranting further studies.

### The Mediating Role of Paper Folding Performance

This study found that the BFS played the mediating role between PI and early SA. This finding has highlighted the important role of PFA in promoting early SA, providing empirical evidence to support the new trend to treat the PFA as a learning context for EEE (Taylor and Hutton, 2013; Lippard et al., 2019). Also, the finding that PFA could predict early SA has provided empirical evidence to support Taylor and Hutton (2013) to promote STEM education through implementing folding activity in early childhood settings. However, this study also found the AFS did not play any roles in the relationship between PI and Early Spatial Ability. This finding indicated that PI could only predict young children’s BFSs, thus indirectly facilitating their SA. In addition, this study found that PI could not predict the AFS, indicating that these skills might be influenced by other confounding factors, such as cognitive level, which is more genetic-oriented thus could not be facilitated by the ‘nurturing’ measures. Therefore, well-designed experimental or large-scale longitudinal studies should be conducted in the future to confirm the cause-effect relationships between them and to evaluate the intervention effects. This study, however, can only confirm the predictive relationship using the cross-sectional data.

## Conclusion, Limitations, and Implications

This study has achieved the following conclusions. First, significant age differences were found in the PFP and early SA, indicating that both of them were still developing in the early years. No significant age differences were found in the BFSs between the 4-year-old and 5-year-old children. Second, no significant gender differences were found in the PFP and early SA, challenging the belief that there are gender differences in Chinese children’s SA. Third, PI could significantly contribute to the BFS level of the paper folding task in Chinese preschoolers. Fourth, paper folding skills could significantly predict SA after controlling for age, gender, SES, parental education levels, and PI. Last, BFSs played a mediating role in the relationship between PI and early SA.

This study, however, has some limitations. First, a cross-sectional study cannot explore the cause-effect relationships between PI and children’s SA. Well-designed experimental or large-scale longitudinal studies should be conducted in the future to confirm the causality. Second, the paper folding task was newly developed and validated in this study, and the map-use task was adapted from Bluestein and Acredolo (1979). They should be further validated by a large-scale sample in the future.

Nevertheless, this study has some implications for future directions and parental education. First, the finding that there were predictive relationships between PI, paper folding, and SA implies that PFA might potentially facilitate the development of spatial abilities thus deserves further studies. Second, the finding that there were no significant age differences in the BFSs between the Age 4 and Age 5 groups implies that more attention should be paid to younger children’s training under Age 4. Third, the finding that there were no significant gender differences in paper folding and map-use implies that the traditional stereotype about gender difference should be abandoned, and early childhood education should not be gendered. Last but not least, the finding that PI might have an indirect impact on early spatial development implies that parental education programs should consider including the promotion and training of paper folding skills. This is especially convenient and workable in the contexts with well-established family kindergarten partnerships, such as in Hong Kong (Jeynes, 2006; Lau et al., 2012).

## Data Availability Statement

The raw data supporting the conclusions of this article will be made available by the authors, without undue reservation.

## Ethics Statement

The studies involving human participants were reviewed and approved by the Ethical Committee of the Education University of Hong Kong. Written informed consent to participate in this study was provided by the participants’ legal guardian/next of kin.

## Author Contributions

DW and JS designed the study and drafted the whole manuscript together. DW collected and analyzed the data under the supervision of JS. Both authors contributed to the article and approved the submitted version.

## Conflict of Interest

The authors declare that the research was conducted in the absence of any commercial or financial relationships that could be construed as a potential conflict of interest.

## References

[B1] Alebna V. Akayuure P. Asiedu-Addo S. K. (2016). Investigating the effect of origami instruction on preservice teachers’ spatial ability and geometric knowledge for teaching. *Int. J. Educ. Math. Sci. Technol.* 4 198–209. 10.18404/ijemst.78424

[B2] Arsl O. Işıksal-Bostan M. (2016). Turkish prospective middle school mathematics teachers’ beliefs and perceived self-efficacy beliefs regarding the use of origami in mathematics education. *Eurasia J. Math. Sci. Technol. Educ.* 12 1533–1548.

[B3] Astur R. S. Tropp J. Sava S. Constable R. T. Markus E. J. (2004). Sex differences and correlations in a virtual Morris water task, a virtual radial arm maze, and mental rotation. *Behav. Brain Res.* 151 103–115. 10.1016/j.bbr.2003.08.024 15084426

[B4] Blades M. Spencer C. (1986). Map use by young children. *Geography* 71 47–52.

[B5] Bluestein N. Acredolo L. (1979). Developmental changes in map-reading skills. *Child Dev.* 50 691–697. 10.2307/1128934

[B6] Boakes N. J. (2009). Origami instruction in the middle school mathematics classroom: its impact on spatial visualization and geometry knowledge of students. *RMLE Online* 32 1–12. 10.1080/19404476.2009.11462060

[B7] Burte H. Gardony A. L. Hutton A. Taylor H. A. (2017). Think3d!: improving mathematics learning through embodied spatial training. *Cogn. Res. Principles Implications* 2:13. 10.1186/s41235-017-0052-9 PMC5318486 28275706

[B8] Cakmak S. Isiksal M. Koc Y. (2014). Investigating the effect of origami-based instruction on elementary students’ spatial skills and perceptions. *J. Educ. Res.* 107 59–68. 10.1080/00220671.2012.753861

[B9] Casey M. B. (1996). Understanding individual differences in spatial ability within females: anature/nurture interactionist framework. *Dev. Rev.* 16 241–260. 10.1006/drev.1996.0009

[B10] Castro M. Expósito-Casas E. López-Martín E. Lizasoain L. Navarro-Asencio E. Gaviria J. L. (2015). Parental involvement on student academic achievement: a meta-analysis. *Educ. Res. Rev.* 14 33–46. 10.1016/j.edurev.2015.01.002

[B11] Chan D. W. (2007). Gender differences in spatial ability: relationship to spatial experience among Chinese gifted students in Hong Kong. *Roeper Rev.* 29 277–282. 10.1080/02783190709554423

[B12] Dinehart L. Manfra L. (2013). Associations between low-income children’s fine motor skills in preschool and academic performance in second grade. *Early Educ. Dev.* 24 138–161. 10.1080/10409289.2011.636729

[B13] Fan X. Chen M. (2001). Parental involvement and students’ academic achievement: a meta-analysis. *Educ. Psychol. Rev.* 13 1–22.

[B14] Freundschuh S. (1990). Can young children use maps to navigate? *Cartographica* 27 54–66. 10.3138/483m-2n11-t168-7436

[B15] Gilmartin P. P. Patton J. C. (1984). Comparing the sexes on spatial abilities: map-use skills. *Ann. Assoc. Am. Geogr.* 74 605–619. 10.1111/j.1467-8306.1984.tb01477.x

[B16] Gutman L. M. Feinstein L. (2010). Parenting behaviors and children’s development from infancy to early childhood: changes, continuities, and contributions. *Early Child Dev. Care* 180 535–556. 10.1080/03004430802113042

[B17] Halpern D. F. (1992). *Sex Differences in Cognitive Abilities*, 2nd Edn Hillsdale, NJ: Erlbaum.

[B18] Harris J. Hirsh-Pasek K. Newcombe N. S. (2013). Understanding spatial transformations: similarities and differences between mental rotation and mental folding. *Cogn. Process.* 14 105–115. 10.1007/s10339-013-0544-6 23397105

[B19] Harte D. Spencer K. (2014). Sleight of hand: magic, therapy and motor performance. *J. Hand Ther.* 27 67–69. 10.1016/j.jht.2013.11.001 24373452

[B20] Hoffman M. Gneezy U. List J. A. (2011). Nurture affects gender differences in spatial abilities. *Proc. Natl. Acad. Sci. U.S.A.* 108 14786–14788. 10.1073/pnas.1015182108 21876159 PMC3169128

[B21] Ilen L. (2016). *Mental Rotation and Mental Folding in 7-and 8-Year-Old Children.* Master’s Thesis, University of Jyväskylä, Jyväskylän.

[B22] Imaroonrak S. Phunwutikorn P. Phattharayuttawat S. Ngamthipwatthana T. Sumalrot T. Auampradit N. (2018). The effects of origami training on creativity and visual-motor integration in preschool children. *J. Med. Assoc. Thailand* 101 S85–S89.

[B23] Jeynes W. (2006). Standardized tests and froebel’s original kindergarten model. *Teach. College Rec.* 108 1937–1959. 10.1111/j.1467-9620.2006.00769.x

[B24] Jeynes W. H. (2007). The relationship between parental involvement and urban secondary school student academic achievement: a meta-analysis. *Urban Educ.* 42 82–110. 10.1177/0042085906293818

[B25] Kass S. J. Ahlers R. H. Dugger M. (1998). Eliminating gender differences through practice in an applied visual-spatial task. *Hum. Perform.* 11 337–349. 10.1207/s15327043hup1104_3

[B26] Kuhl P. K. Lim S. S. Guerriero S. van Damme D. (2019). *Shapes, Blocks, Puzzles, and Origami: From Spatial Play to STEM Learning. From the Book: Developing Minds in the Digital Age: Towards a Science of Learning for 21st Century Education. Educational Research and Innovation.* France: OECD Publishing.

[B27] Lau E. Y. H. Li H. Rao N. (2012). Exploring parental involvement in early years education in China: development and validation of the Chinese Early Parental Involvement Scale (CEPIS). *Int. J. Early Years Educ.* 20 405–421. 10.1080/09669760.2012.743099

[B28] Levine S. C. Foley A. Lourenco S. Ehrlich S. Ratliff K. (2016). Sex differences in spatial cognition: advancing the conversation. *Wiley Interdiscip. Rev. Cogn. Sci.* 7 127–155. 10.1002/wcs.1380 26825049

[B29] Li H. Chau L. (2010). “Story approach to integrated learning (SAIL): a postmodernism curriculum for Hong Kong kindergartens,” in *Handbook of Curriculum Development*, ed. Kattington L. E. (Hauppauge, NY: Nova Science), 329–346.

[B30] Li H. Rao N. Tse S. K. (2012). Adapting Western pedagogies for Chinese literacy instruction: case studies of Hong Kong, Shenzhen, and Singapore preschools. *Early Educ. Dev.* 23 603–621. 10.1080/10409289.2010.536441

[B31] Liben L. S. Myers L. J. Christensen A. E. Bower C. A. (2013). Environmental-Scale map use in middle childhood: links to spatial skills, strategies, and gender. *Child Dev.* 84 2047–2063. 10.1111/cdev.12090 23550840

[B32] Lippard C. N. Lamm M. H. Tank K. M. Choi J. Y. (2019). Pre-engineering thinking and the engineering habits of mind in preschool classroom. *Early Childhood Educ. J.* 47 187–198. 10.1007/s10643-018-0898-6

[B33] Lomax-Bream L. E. Taylor H. B. Landry S. H. Barnes M. A. Fletcher J. M. Swank P. (2007). Role of early parenting and motor skills on development in children with spina bifida. *J. Appl. Dev. Psychol.* 28 250–263. 10.1016/j.appdev.2007.02.004

[B34] Maeda Y. Yoon S. Y. (2013). A meta-analysis on gender differences in mental rotation ability measured by the Purdue spatial visualization tests: visualization of rotations (PSVT: R). *Educ. Psychol. Rev.* 25 69–94. 10.1007/s10648-012-9215-x

[B35] Milivojevic B. Johnson B. W. Hamm J. P. Corballis M. C. (2003). Non-identical neural mechanisms for two types of mental transformation: event-related potentials during mental rotation and mental paper folding. *Neuropsychologia* 41 1345–1356. 10.1016/s0028-3932(03)00060-5 12757907

[B36] Mix K. S. Levine S. C. Cheng Y.-L. Young C. Hambrick D. Z. Ping R. (2016). Separate but correlated: the latent structure of space and mathematics across development. *J. Exp. Psychol. Gen.* 145 1206–1227. 10.1037/xge0000182 27560854

[B37] Newcombe N. S. (2010). Picture this: increasing math and science learning by improving spatial thinking. *Am. Educ.* 34:29.

[B38] Nishida Y. (2019). Something old, something new, something borrowed, and something Froebel? The development of origami in early childhood education in Japan. *Paedagogica Historica* 55 529–547. 10.1080/00309230.2018.1546330

[B39] Oberman J. (2018). “Origametria—paper folding for teaching geometry in preschool and primary school,” in *K-12 Mathematics Education In Israel: Issues And Innovations*, ed. Movshovitz-Hadar N. (Singapore: World Scientific), 89–95. 10.1142/9789813231191_0009

[B40] Parsons T. D. Larson P. Kratz K. Thiebaux M. Bluestein B. Buckwalter J. G. (2004). Sex differences in mental rotation and spatial rotation in a virtual environment. *Neuropsychologia* 42 555–562. 10.1016/j.neuropsychologia.2003.08.014 14728927

[B41] Peters M. (2005). Sex differences and the factor of time in solving Vandenberg and Kuse mental rotation problems. *Brain Cogn.* 57 176–184. 10.1016/j.bandc.2004.08.052 15708213

[B42] Rimfeld K. Shakeshaft N. G. Malanchini M. Rodic M. Selzam S. Schofield K. (2017). Phenotypic and genetic evidence for a unifactorial structure of spatial abilities. *Proc. Natl. Acad. Sci. U.S.A.* 114 2777–2782. 10.1073/pnas.1607883114 28223478 PMC5347574

[B43] Rittle-Johnson B. Zippert E. L. Boice K. L. (2019). The roles of patterning and spatial skills in early mathematics development. *Early Childhood Res. Q.* 46 166–178. 10.1016/j.ecresq.2018.03.006

[B44] Rubin K. H. Burgess K. B. Hastings P. D. (2002). Stability and social–behavioral consequences of toddlers’ inhibited temperament and parenting behaviors. *Child Dev.* 73 483–495. 10.1111/1467-8624.00419 11949904

[B45] Sandberg E. H. Huttenlocher J. (2001). Advanced spatial skills and advance planning: components of 6-year-olds’ navigational map use. *J. Cogn. Dev.* 2 51–70. 10.1207/s15327647jcd0201_3

[B46] Saraiva L. Santos F. Madronna P. G. Cesar S. Á (2019). “Fine motor skills: an emergent competence in preschool age,” in *Proceedings of the International Seminar of Physical Education, Leisure and Health, 17–19 June 2019*, Castelo Branco.

[B47] Sato F. Shimoyama I. Shibukawa S. (2007). Decision making, working memory, and the effects of learning: a comparative analysis of near-infrared spectroscopy analyses of the frontal lobe and self-reported subject responses. *Chiba Med. J.* 83 1–9.

[B48] Seng A. S. H. Tan L. C. (2002). “Cultural and gender differences in spatial ability of young children,” in *Proceedings of the ACEI Annual Conference*, San Diego, CA.

[B49] Stieff M. Uttal D. (2015). How much can spatial training improve STEM achievement? *Educ. Psychol. Rev.* 27 607–615. 10.1007/s10648-015-9304-8

[B50] Taylor H. A. Hutton A. (2013). Think3d!: training spatial thinking fundamental to STEM education. *Cogn. Instr.* 31 434–455. 10.1080/07370008.2013.828727

[B51] Tenbrink T. Taylor H. A. (2015). Conceptual transformation and cognitive processes in Origami paper folding. *J. Problem Solv.* 8:1.

[B52] Tosto M. G. Hanscombe K. B. Haworth C. M. Davis O. S. Petrill S. A. Dale P. S. (2014). Why do spatial abilities predict mathematical performance? *Dev. Sci.* 17 462–470. 10.1111/desc.12138 24410830 PMC3997754

[B53] Uttal D. H. Meadow N. G. Tipton E. Hand L. L. Alden A. R. Warren C. (2013). The malleability of spatial skills: a meta-analysis of training studies. *Psychol. Bull.* 139:352. 10.1037/a0028446 22663761

[B54] Vidoni C. Lorenz D. J. de Paleville D. T. (2014). Incorporating a movement skill programme into a preschool daily schedule. *Early Child Dev. Care* 184 1211–1222. 10.1080/03004430.2013.856895

[B55] Voyer D. Voyer S. Bryden M. P. (1995). Magnitude of sex differences in spatial abilities: a meta-analysis and consideration of critical variables. *Psychol. Bull.* 117:250. 10.1037/0033-2909.117.2.250 7724690

[B56] Wenciker B. Flynn P. (2004). “Modular origami in the mathematics classroom,” in *Proceedings of the Bridges: Mathematical Connections in Art, Music, and Science Bridges Conference* (Los Angeles: Bridges).

[B57] Widayati S. Simatupang N. D. Sari P. P. (2019). “The impact of adduction of folding paper stages for children’s fine motor skills,” in *Proceedings of the 3rd International Conference on Education Innovation (ICEI 2019)*, (Paris: Atlantis Press).

[B58] Wright R. Thompson W. L. Ganis G. Newcombe N. S. Kosslyn S. M. (2008). Training generalized spatial skills. *Psychon. Bull. Rev.* 15 763–771. 18792502 10.3758/pbr.15.4.763

[B59] Yao W. Dai J. S. (2008). Dexterous manipulation of origami cartons with robotic fingers based on the interactive configuration space. *J. Mech. Des.* 130:022303.

[B60] Yuzawa M. Bart W. M. (2002). Young children’s learning of size comparison strategies: effect of origami exercises. *J. Genet. Psychol.* 163 459–478. 10.1080/00221320209598696 12495231

[B61] Yuzawa M. Bart W. M. Kinne L. J. Sukemune S. Kataoka M. (1999). The effect of “origami” practice on size comparison strategy among young Japanese and American children. *J. Res. Childhood Educ.* 13 133–143. 10.1080/02568549909594734

[B62] Zhang D. (2017). Effects of visual working memory training and direct instruction on geometry problem solving in students with geometry difficulties. *Learn. Disabil. Contemp. J.* 15 117–138.

[B63] Zhao X. (2015). Current situation analysis and educational implications of origami activities among Chinese children of different ages. *J. Educ. Dev.* 2 33–35.

